# Accurate evaluation of combustion enthalpy by ab-initio computations

**DOI:** 10.1038/s41598-022-09844-z

**Published:** 2022-04-06

**Authors:** Amin Alibakhshi, Lars V. Schäfer

**Affiliations:** 1 Theoretical Chemistry, Institute for Physical Chemistry, Christian-Albrechts-University, Olshausenstr. 40, 24118 Kiel, Germany; 2 Theoretical Chemistry, Ruhr University Bochum, 44780 Bochum, Germany

**Keywords:** Chemistry, Chemical safety, Physical chemistry, Theoretical chemistry

## Abstract

Accurate evaluation of combustion enthalpy is of high scientific and industrial importance. Although ab-initio computation of the heat of reactions is one of the promising and well-established approaches in computational chemistry, reliable and precise computation of heat of combustion reactions by ab-initio methods is surprisingly scarce in the literature. A handful of works carried out for this purpose report significant inconsistencies between the computed and experimentally determined combustion enthalpies and suggest empirical corrections to improve the accuracy of the ab-initio predicted data. The main aim of the present study is to investigate the reasons behind those reported inconsistencies and propose guidelines for a high-accuracy estimation of heat of reactions via ab-initio computations. We show comparably accurate prediction of combustion enthalpy of 40 organic molecules based on a DSD-PBEP86 double-hybrid density functional theory approach and CCSD(T)-F12 coupled-cluster computations, with mean unsigned errors with respect to experimental data being below 0.5% for both methods.

## Introduction

Combustion is the key process in many important scientific and industrial applications such as power production, transportation, heating, synthesis, and processing of materials^1^. Despite being an active research area for over a century, fully understanding—let alone predicting—many aspects of combustion processes is still a scientific challenge^2^. Unraveling these challenges sometimes requires research under special operational conditions, e.g., combustion experiments under microgravity conditions, which is one of the ongoing research activities in the international space station^3^. Alongside the experimental researches, theoretical studies have also substantially contributed to unraveling many of the complexities in combustion science. High-level quantum-mechanical computations have been found to be a highly promising tool for studying the kinetics of combustion reactions^4–9^, elucidating the chemical pathways^10–12^, or studying combustion thermochemistry^13–15^.

Several advanced methods such as Feller–Peterson–Dixon^16^, focal point^17^, HEAT^18^, and W4^19^ have been proposed in the recent years for highly precise computation of the heat of combustion reactions. Nevertheless, examples of successful applications of traditional methods in ab-initio computations leading to high-accuracy prediction of combustion enthalpy are surprisingly scarce in the literature. In a few studies carried out for this purpose so far, substantial inconsistencies have been reported between the theoretically predicted and experimentally determined combustion enthalpies, as discussed in the following.

Whyman et al.^20^ employed MP2 ab-initio computations to evaluate the combustion enthalpies and heats of formation of 31 compounds. Although they have not reported the accuracy of their theoretically calculated combustion enthalpies in comparison with the experimental data, their computations demonstrate significant deviations compared to the experiment, as we discussed below. Audran and co-workers^21^ employed ab-initio computations to calculate combustion enthalpies using four different levels of theory. They reported significant deviations between the computational and experimental data and proposed linear relationships to empirically improve the theoretically predicted combustion enthalpies. Mazzuca et al.^22^ studied seven ab-initio methods for evaluating the combustion enthalpies of 31 compounds. To reduce the observed deviations with the experimental combustion enthalpies, they suggested empirical scaling factors ranging from 0.9846 to 1.1866, depending on the employed level of theory. This empirical scaling, however, did not improve the mean unsigned errors to below 3%.

Considering that evaluation of enthalpies of chemical reactions by conventional quantum mechanical computations is one of the most widely benchmarked and well-established applications of quantum chemistry, the issues described above need to be addressed. Therefore, the present study investigates the reasons behind the reported deviations between the theoretically computed and experimentally determined enthalpies of combustion reactions. We provide insights into the error sources, propose a treatment, and suggest guidelines for high accuracy determination of combustion enthalpies by quantum chemical computations.

## Theoretical evaluation of combustion enthalpies

Precise evaluation of enthalpies of gas-phase reactions is commonly achieved via ab-initio computation of enthalpies for the individual molecules involved in the reactions. Nevertheless, when it comes to combustion reactions, considering that the reactants and products might not always be in the gaseous state, phase change thermodynamics can also play an important role and should be taken into account, which significantly adds to the computational challenges. Such phase change enthalpies are often not considered, which can be one of the main contributions to inconsistencies between theoretical and experimental combustion enthalpies, as shown in the results section.

For appropriate treatment of phase change thermodynamics in combustion reactions, careful attention has to be paid to differences in defining the state of reactants and products and to different conventions in reporting combustion enthalpies. The experimentally determined combustion enthalpy data, commonly measured by oxygen bomb calorimetry, are typically reported either as gross or net heat of combustion. Gross heat of combustion refers to the total amount of heat released in the calorimetry experiment, where both the reactants and products are at room temperature and in their standard states^23^. In contrast, for the net heat of combustion, while the reactants are considered in their standard states, the combustion products are assumed to be in the gas phase^23^. Clearly, the most obvious deviation between the reported gross and net heats of combustion is due to the heat released by the condensation of water molecules produced in the combustion reaction, which occurs as a result of cooling down the combustion products by the heat bath in which the combustion chamber is placed during the calorimetry experiment.

In addition to phase change thermodynamics, inaccuracies in both the theoretical methods as well as the experiments can significantly influence the accuracy of the obtained results. Among them, the accuracy of the level of theory employed in the quantum chemical calculations plays a key role. In addition, the results might critically depend on whether or not the relevant minimum-energy conformers have been identified. The main aim of the present study is to investigate and benchmark the impact of these intricacies and demonstrate the employment of ab-initio quantum chemical computations for achieving high accuracy prediction of combustion enthalpy.

## Computational details

The experimentally determined gross heats of combustion for 40 organic molecules reported by Walters^24^ were used as reference combustion enthalpies. The full list of the studied hydrocarbons is reported in Table 1. The selected molecules only contain C, H, and O atoms, to avoid complications e.g. due to solvation of nitric or sulfuric acid in water, which are produced by combustion of molecules containing nitrogen or sulfur and leading to contributions to the measured combustion enthalpy^23^.

**Table 1:** Details of the theoretical and experimental data.

Compound	Std.	ΔH_std-gas_	H_QM, reactant (CCSD(T)-F12b)_	ΔH_QM,comb.(CCSD(T) -F12b)_	H_QM, reactant (DSD-PBEP86)_	ΔH_QM,comb. (DSD-PBEP86)_	ΔH_exp_
Oxirane	g	0	− 403,138.66	− 1309.47	− 403,132.4	− 1310.65	− 1305.53
Cyclopentane	l	28.8	− 514,933.78	− 3302.86	− 514,947.83	− 3302.17	− 3288.85
Ethylbenzene	l	41	− 814,591.38	− 4587.09	− 814,646.33	− 4593.82	− 4561.44
2-Butanone	l	34	/	/	− 609,289.96	− 2453.09	− 2442.95
Methanol	l	37.6	− 303,407.35	− 726.84	− 303,387.42	− 725.58	− 726.47
Cyclobutane	g	0	− 411,836.49	− 2752.82	− 411,847.33	− 2752.67	− 2742.09
Acetone	l	31.27	− 506,298.95	− 1796.51	− 506,295.82	− 1797.23	− 1789.6
Dimethyl ether	g	0	− 612,346.35	− 2729.83	− 612,335.26	− 2727.75	− 1459.71
2-Propanol	l	45	− 509,417.59	− 2011.25	− 509,403.14	− 2009.87	− 2004.92
Ethane	g	0	− 209,065.2	− 1562.84	− 209,060.73	− 1559.23	− 1558.59
Acetaldehyde	g	0	− 403,251.43	− 1196.7	− 403,245.03	− 1198.02	− 1191.93
Cyclopropane	g	0	− 308,842.15	− 2099.83	− 308,851.07	− 2098.93	− 2089.79
Formic acid	l	46.3	− 497,700.77	− 253.51	− 497,680.78	− 255.3	− 254.46
Ethanol	l	42.3	− 406,411.32	− 1370.2	− 406,394.31	− 1368.7	− 1366.23
Butane	g	0	− 415,037.08	− 2885.62	− 415,038.59	− 2881.36	− 2874.96
Ethyl acetate	l	35	− 806,651.63	− 2244.63	− 806,639.85	− 2246.24	− 2237.68
Isopropyl benzene	l	44	− 917,586.63	− 5239.17	− 917,644.63	− 5245.52	− 5212.17
Diethyl ether	l	27.1	/	/	− 406,302.41	− 1460.59	− 2722.42
Benzene	l	33.9	− 608,599.98	− 3283.83	− 608,647.31	− 3292.83	− 3264.75
1,4-Dioxane	l	38	− 806,529.49	− 2366.76	− 806,515.02	− 2371.07	− 2362.73
1,2-Ethanediol	l	65	− 603,743.92	− 1191.07	− 603,714.14	− 1191.9	− 1189.44
Phenol	s	69.7	− 805,966.71	− 3070.57	− 806,003.58	− 3079.61	− 3051.84
Vinyl acetate	l	37.2	− 803,475.5	− 2087.37	− 803,470.61	− 2095.52	− 2080.62
Propanol	l	47	− 509,402.47	− 2026.37	− 509,388.38	− 2024.62	− 2018.73
Heptane	l	36	− 724,029.45	− 4835.23	− 724,039.69	− 4830.27	− 4813.15
Cyclohexane	l	33.1	− 617,950.83	− 3933.14	− 617,968.58	− 3931.43	− 3917.19
1-Pentanol	l	57	− 715,380.89	− 3342.61	− 715,372.74	− 3340.26	− 3328.86
Glycerol	l	91.7	− 904,085.23	− 1650.56	− 904,045.12	− 1653.97	− 1652.52
Propane	g	0	− 312,049.9	− 2225.47	− 312,048.46	− 2221.49	− 2218.62
Acetic acid	l	50.3	− 600,722.44	− 879.17	− 600,705.37	− 880.72	− 874.05
Pentane	l	26.5	− 518,050.9	− 3519.13	− 518,055.32	− 3514.64	− 3506.75
Isopropyl ether	l	32.26	− 818,350.64	− 4020.18	− 818,344.04	− 4018.96	− 4008.9
Furan	l	27.71	− 602,983.78	− 2092.23	− 603,001.12	− 2102.02	− 2082.44
Toluene	l	37	− 711,599.57	− 3931.57	− 711,651.16	− 3938.99	− 3906.28
Hexane	l	31	− 621,042.54	− 4174.81	− 621,049.85	− 4170.11	− 4160.07
1-Methylnaphthalene	l	59	− 1,114,276.48	− 5843.82	− 1,114,373.98	− 5856.3	− 5808.23
Benzaldehyde	l	48	− 905,806.66	− 3544.57	− 905,857.39	− 3555.84	− 3526.08
Cyclohexene	l	33.57	− 614,783.85	− 3766.73	− 614,808.73	− 3771.33	− 3748.59
1-Butene	g	0	− 411,861.29	− 2728.02	− 411,869.07	− 2730.93	− 2715.74
m-Cresol	l	60	− 908,954.1	− 3730.51	− 908,995.09	− 3738.1	− 3702.26
AAD				11.94		13.29	
MUE%				0.40%		0.44%	

All enthalpies are in kJ/mol.

The columns from left to right represent: *Std.* standard state (gas = g, liquid = l, solid = s), *ΔH*_*std-gas*_ the enthalpy of phase change from the standard state to the gas phase, *H*_*QM,reactant*_ the QM enthalpies of individual reactants in the gas phase, *ΔH*_*QM,comb*._ the combustion enthalpy directly obtained via QM enthalpy of reaction, *ΔH*_*exp*_ the experimentally determined data.

Computation of in-vacuo enthalpies of compounds was carried out by normal mode analysis based on the rigid rotor harmonic oscillator approximation^25^. To that end, for each compound, the geometries of the molecules were optimized in vacuo. These optimized structures were then used to calculate the ground-state electronic energies and normal mode vibrational frequencies required for calculating thermal effects. The computed enthalpies were then corrected for hindered rotation based on Truhlar’s method^26^.

The molecular configurations found at this stage by geometry optimization can yield a wide range of energies, and thus the details of the geometries found can play a significant role in the ab-initio-evaluated molecular enthalpies and hence the resulting combustion enthalpies. To identify the conformers corresponding to the (global) minima on the energy landscape, a systematic conformer search was carried out using 20 different initial structures generated via the genetic algorithm module of the openbabel toolbox^27^. The configuration which yielded the lowest energy after optimization was then used for the calculation of the combustion enthalpy.

Quantum-mechanical (QM) computations were carried out at the DSD-PBEP86-D3/def2-QZVP level of theory, an accurate double-hybrid density functional theory (DFT) method with a computational cost that is comparable to MP2 computations^28–30^. The employed quadruple-zeta basis set is large enough for standard DFT usage and considering that DFT is commonly assumed to be converged with a triple-zeta basis set, basis-set errors are likely very small. Additionally, one of the largest contributors to DFT inaccuracy, which is due to van-der-Waals interactions, is taken care of by Grimme’s well-tested D3 correction. For the geometries optimized by DSD-PBEP86-D3/def2-QZVP, the electronic ground state energies were also computed at the explicitly correlated CCSD(T)-F12b/def2-QZVP coupled cluster level of theory. Additionally, to further investigate the influence of the selected density functional, the molecular enthalpies were also computed at the B3LYP/6-311+G(2d,p) level of theory, which is one of the most widely used hybrid functionals.

Considering that the theoretically computed enthalpies are for molecules in vacuo, for non-gaseous reactants, the QM evaluated enthalpy of the reactants in their standard state was estimated by subtracting their heat of phase change from the standard state to the gas phase from the initially computed in-vacuo enthalpies. Although for that purpose, the thermodynamic quantities in the condensed phase can be theoretically evaluated either with implicit solvent approaches^31^, statistical thermodynamics models^32^ or empirically applied through machine learning^33^, a more straightforward way to take them into account is via experimental phase change data. To that end, the phase change enthalpies were taken from the NIST database. Similarly, considering that the reference data used in the present study are gross heats of combustion, the vaporization enthalpy of water with the value of 42.773 kJ/mol, as recommended by the ASTM method^23^, was subtracted from the QM evaluated enthalpy of water in vacuo to yield the QM enthalpy of water in the liquid state. The enthalpy of O_2_ was calculated for the triplet spin multiplicity as the ground electronic state^20^.

Since measurement of combustion enthalpies is commonly carried out under 30 bar pressure^23^, we also investigated the pressure impacts on the enthalpy. To that end, we exploited the following thermodynamic relationship:

1 $$\left( {\frac{\partial H}{{\partial P}}} \right)_{T} = V + T\left( {\frac{\partial S}{{\partial P}}} \right)_{T} = V - T\left( {\frac{\partial V}{{\partial T}}} \right)_{P} .$$

Considering that the changes in thermal expansion of solids and liquids for increasing the pressure from 1 to 30 bar is negligible, we only evaluated the pressure impacts on the enthalpies of gaseous compounds. To that end, the molar volume of gaseous compounds and their derivative with respect to temperature were evaluated via the Redlich–Kwong equation of state defined as^34^:

2 $$P = \frac{RT}{{V - b}} - \frac{a}{{\sqrt T V \left( {V + b} \right)}},$$

3 $$a = 0.42748 \frac{{R^{2} T_{c}^{2.5} }}{{P_{c} }},$$

4 $$b = 0.08664\frac{{R T_{c} }}{{P_{c} }},$$

where $R$ is the universal gas constant, $V$ is the molar volume, and $T_{c}$ and $P_{c}$ are the critical temperature and pressure, respectively. Accordingly, for pressures from 1 to 30 bar with 1 bar intervals, the molar volumes were calculated for temperatures from 280 to 320 K with 1 K intervals via solving Eq. (2) with the bisection method. Using the calculated V-T values for each pressure, a third-order polynomial was fitted and used to calculate the partial derivative $\frac{\partial V}{{\partial T}}$ in Eq. (1). Using the calculated molar volumes and $\frac{\partial V}{{\partial T}}$ for all pressures, the pressure-induced enthalpy changes were calculated by numerically evaluating the following integral:

5 $$\Delta H = \mathop \smallint \limits_{1}^{30} \left( {V - T\left( {\frac{\partial V}{{\partial T}}} \right)_{P} } \right)dp.$$

The accuracies of the computationally predicted combustion enthalpies with respect to the experimental data are reported as average absolute deviation (AAD) and percentage mean unsigned error (MUE%), defined as:

6 $$AAD = \frac{1}{N} \sum \left( {\left| {y_{i}^{exp} - y_{i}^{pred} } \right|} \right),$$

7 $$MUE\% = \frac{1}{N} \sum \left( {\left| {\frac{{y_{i}^{exp} - y_{i}^{pred} }}{{y_{i}^{exp} }}} \right|} \right) \times 100.$$

The DFT and CCSD(T)-F12 computations were carried out with the Gaussian16^35^ and Molpro^36^ quantum chemistry packages, respectively.

## Results and discussion

The details of the computed molecular enthalpies at the different levels of theory employed in this work are reported in Table 1, together with the experimental values.

**Figure 1:**
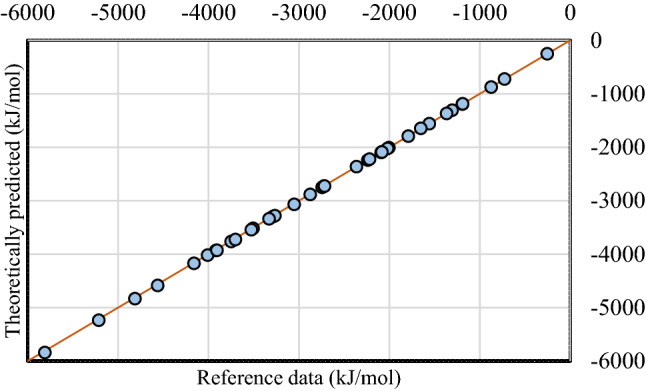
Comparison of theoretically predicted and experimentally determined combustion enthalpies. The data shown are from the CCSD(T)-F12 computations, because the DSD-PBEB86 values are visually indistinguishable on the plotted range of enthalpy values.

Using the QM-evaluated enthalpies corrected for phase change enthalpies of water and reactants, the predicted combustion enthalpies yielded AAD, MUE% and correlation coefficient of 11.94 kJ/mol, 0.40%, and 0.99999, respectively, for the CCSD(T)-F12b computations, and 13.29 kJ/mol, 0.44%, and 0.99998, respectively, for the DSD-PBEB86 computations. A comparison of the computed and reference combustion enthalpies are depicted in Fig. 1.

These results, which are directly obtained by ab-initio computation without any empirical correction, show a remarkable improvement compared to results reported in previous studies. For example, the theoretically calculated combustion enthalpies reported by Mazzuca et al.^22^ yielded a MUE of roughly 3%, even after applying an empirical scaling. According to the results, taking into account the high pressure impacts the combustion enthalpy only marginally, and yields an improved AAD of predicted results by only 0.01 kJ/mol. Similarly, the hindered rotor correction improves the AAD of the predicted combustion enthalpies by only 0.088 kJ/mol.

The results reported in Table 1 show that the accuracy of the employed level of theory plays a key role. To further demonstrate the importance of the applied level of theory, we also computed the combustion enthalpies at the B3LYP/6-311+G(2d,p) level of theory for the same set of molecules. The B3LYP/6-311+G(2d,p) computations yielded AAD, MUE%, and correlation coefficient of 104.35 kJ/mol, 3.93%, and 0.9988, respectively, which are roughly one order of magnitude less accurate than those obtained with DSD-PBEP86-D3/def2-QZVP or CCSD(T)-F12b/def2-QZVP.

Analyzing the computed energies shows that molecular thermal energies, i.e. the kinetic energy due to rotation/translation and vibrational energies, contribute on average only 0.625% and 0.541% to the computed combustion enthalpies, and the changes in ground state electronic energies of reactants and products are the main contributions to the heat released by combustion. Thus, the accuracy of the employed level of theory in reproducing the ground state electronic energy plays the key role for the accuracy of the obtained results. For the electronic energies evaluated with DSD-PBEP86-D3/def2-QZVP and B3LYP /6-311+G(2d,p), we observed an AAD of 148.425 kJ/mol between the ground state electronic energies obtained with these two DFT methods, while for thermal energies the AAD was only 0.781 kJ/mol. These results also reveal why the accuracy of theoretical methods for combustion reactions is so different from the benchmark results obtained for other case studies. The reason is that the large amount of energy released by combustion reactions is mainly due to electronic energies, which implies substantial differences between electronic energies of reactants and products.

The DSD-PBEP86-D3/def2-QZVP level of theory used in the present study supersedes most of the conventionally accepted functionals in studying thermochemistry^28^. In comparison to the computations by the computationally much more demanding CCSD(T)-F12 computations, which are considered to be a gold standard in theoretical chemistry^37^, DSD-PBEP86-D3 yields only marginally (by 0.04%) lower accuracy in the predicted combustion enthalpies, and therefore provides an excellent cost-efficiency relationship.

Next to the accuracy of the employed QM level of theory, another important source of inaccuracy in theoretically evaluated combustion enthalpies can arise from the usage of high-energy conformers instead of the global minimum-energy structure. As for almost all poly-atomic molecules, several local minima exist on the potential energy surface, and thus geometry optimizations started from different initial structures can result in quite diverse conformers and energies, and consequently in different computed combustion enthalpies. As an example, our theoretical computations on the two locally optimized structures of acetic acid, corresponding to different orientations of the hydroxyl proton relative to the second oxygen atom of the carboxylate group (inward- versus outward-pointing) yield quite different combustion enthalpies. While the low-energy structure yields a combustion enthalpy with 8.64 kJ/mol absolute error, the same computation for the higher-energy structure deviates from the experimental value by 29.76 kJ/mol. Inaccuracies from such high-energy conformers can be avoided by employing efficient general global optimization algorithms or rotamer searches^38^ or, for small molecules, using a systematic conformer search via multi-start optimization, as was done in the present study.

Yet another reason of deviation between the QM predicted and optimum enthalpies can be overlooking non-ideality effects. As discussed earlier, increasing the ambient pressure can directly influence the phase change and gas phase enthalpies, while QM enthalpies are computed for molecules in vacuo. We studied the impact of pressure on gas phase enthalpies via Eq. (5). However, this correction was found to only marginally improve the accuracy of the predicted combustion enthalpies, as can be seen in Table 1. The more significant impact of the ambient pressure on gas phase enthalpies can be attributed to the formation of molecular clusters in the gas phase at high pressures. For example, for accurate evaluation of the phase change enthalpy and the saturation vapor pressure of water, it has been shown that clustering of molecules in the gas phase should be taken into account^39^. Such gas phase clustering reduces the gas phase enthalpy compared to the in vacuo state. Similarly, partial condensation of water molecules^23^ as well as dissolution of CO_2_ in the water produced in the combustion process or formation of combustion side-products other than CO_2_ can result in further deviations between ab-initio computation and experiment. One empirical way to take such effects into account is scaling the enthalpies of H_2_O or CO_2_ or both. Accordingly, we found the optimal scaling factor of 0.9999857 for empirically correcting the theoretically predicted enthalpy of water in CCSD(T)-F12b computations, which reduced the AAD (MUE%) to 5.80 kJ/mol (0.26%). Scaling the enthalpy of CO_2_ computed by CCSD(T)-F12b by 0.999994328 even reduces the AAD (MUE%) of predicted combustion enthalpies to 2.64 kJ/mol (0.15%). Yet further improvement of the results can be achieved by simultaneously scaling the enthalpies of H_2_O and CO_2_ computed by CCSD(T)-F12b by 1.000006465 and 0.999992212, which yields AAD (MUE%) of 2.00 kJ/mol (0.12%). These scaling factors are derived from the enthalpies evaluated at the CCSD(T)-F12b level of theory, which might necessitate their re-evaluation for other levels of theory. However, we speculated that, at least for methods that provide results similar to CCSD(T)-F12b, the scaling factors might not strongly depend on the level of theory applied. Indeed, using the (unchanged) scaling factors obtained by CCSD(T)-F12b for the enthalpies computed with DSD-PBEP86-D3/def2-QZVP yields similar improvements, with AAD (MUE%) values of 8.69 kJ/mol (0.33%), 5.21 kJ/mol (0.21%), and 3.70 kJ/mol (0.15%), obtained via scaling the computed enthalpies of H_2_O, CO_2_, and both of them simultaneously, respectively.

In addition to the inaccuracies resulting from the theoretical computations, systematic or operational errors in experimental data can also contribute to inconsistency between the theoretical and experimental reference data. For example, we observed 1.59 kJ/mol AAD in phase change enthalpies of our studied reactants between the NIST and DIPPR databases, which results in the same deviation between the theoretically predicted gross combustion enthalpies calculated using each one of these two databases. Similar to the vaporization enthalpy, the experimentally determined combustion enthalpies from different sources also show some variations. For example, slight inaccuracy in measuring the combustion enthalpy of benzoic acid, which is used to calibrate the calorimeter^23^, can result in a linearly distributed deviation (offset) between measured combustion enthalpies of all other compounds. That can be a potential reason for the suitability of a linear fitting to empirically correct the predicted combustion enthalpies, proposed in several studies^21,22^.

In summary, in the present study, we discuss ab-initio quantum chemistry approaches capable of providing highly accurate predictions of combustion enthalpy. To that end, the main considerations in theoretical computations should be directed towards selecting an appropriate level of theory for the quantum chemistry method applied, and carefully identifying the minimum-energy conformers. For reproducing the net heat of combustion, the phase change enthalpy of the reactants should be subtracted from the QM-evaluated gas-phase enthalpies. For the gross heat of combustion, the vaporization enthalpy of water should also be subtracted from the QM-evaluated gas-phase enthalpy of water. Accordingly, taking the phase change enthalpies, as well as the experimental measurement of combustion enthalpy, into consideration or not can also contribute to inconsistencies between the theoretically predicted and experimentally determined combustion enthalpies.
